# Multi-IsnadSet MIS for Sahih Muslim Hadith with chain of narrators, based on multiple ISNAD

**DOI:** 10.1016/j.dib.2024.110439

**Published:** 2024-04-23

**Authors:** Aziz Mehmood Farooqi, Rauf Ahmed Shams Malick, Muhammad Shahzad Shaikh, Adnan Akhunzada

**Affiliations:** a College of Computing and Information Sciences PAF Karachi Institute of Economics and Technology, Karachi, Pakistan; b Department of Computer Science, FAST-National University of Computer and Emerging Sciences, Karachi 75300, Pakistan; c Department of Sciences and Humanities, FAST-National University of Computer and Emerging Sciences, Karachi 75300, Pakistan; d College of Computing & IT, University of Doha for Science and Technology, P.O. Box 24449, Doha, Qatar; e Al-Ghazali University, Karachi, Sindh, Pakistan

**Keywords:** Multi-Isnad of Hadith Narrators dataset, Chain of narrators, Machine learning, Graph database, Spatial–Temporal data, Social-Network Analysis (SNA), Graph Neural Networks (GNN)

## Abstract

In the Islamic domain, Hadiths hold significant importance, standing as crucial texts following the Holy Quran. Each Hadith contains three main parts: the ISNAD (chain of narrators), TARAF (starting part, often from Prophet Muhammad), and MATN (Hadith content). ISNAD, a chain of narrators involved in transmitting that particular MATN. Hadith scholars determine the trustworthiness of the transmitted MATN by the quality of the ISNAD. The ISNAD's data is available in its original Arabic language, with narrator names transliterated into English.

This paper presents the Multi-IsnadSet (MIS), that has great potential to be employed by the social scientist and theologist. A multi-directed graph structure is used to represents the complex interactions among the narrators of Hadith. The MIS dataset represent directed graph which consists of 2092 nodes, representing individual narrators, and 77,797 edges represent the Sanad-Hadith connections. The MIS dataset represents multiple ISNAD of the Hadith based on the Sahih Muslim Hadith book. The dataset was carefully extracted from online multiple Hadith sources using data scraping and web crawling techniques tools, providing extensive Hadith details. Each dataset entry provides a complete view of a specific Hadith, including the original book, Hadith number, textual content (MATN), list of narrators, narrator count, sequence of narrators, and ISNAD count. In this paper, four different tools were designed and constructed for modeling and analyzing narrative network such as python library (NetworkX), powerful graph database Neo4j and two different network analysis tools named Gephi and CytoScape. The Neo4j graph database is used to represent the multi-dimensional graph related data for the ease of extraction and establishing new relationships among nodes. Researchers can use MIS to explore Hadith credibility including classification of Hadiths (Sahih=perfection in the Sanad/Dhaif=imperfection in the Sanad), and narrators (trustworthy/not). Traditionally, scholars have focused on identifying the longest and shortest Sanad between two Narrators, but in MIS, the emphasis shifts to determining the optimum/authentic Sanad, considering narrator qualities. The graph representation of the authentic and manually curated dataset will open ways for the development of computational models that could identify the significance of a chain and a narrator. The dataset allows the researchers to provide Hadith narrators and Hadith ISNAD that could be used in a wide variety of future research studies related to Hadith authentication and rules extraction. Moreover, the dataset encourages cross-disciplinary research, bridging the gap between Islamic studies, artificial intelligence (AI), social network analysis (SNA), and Graph Neural Network (GNN).

Specifications Table

**Table:** 

Subject	Computer Science.
Specific subject area	Islamic Hadith corpus, Multi-Isnad of Hadith Narrators, Social Network Analysis (SNA), Graph Neural Network (GNN). Neo4j Graph Database
Type of data	Text file, Table, Graph etc. Filtered, Processed etc.
How to data were acquired	The dataset was collected from Hadith websites (these Hadiths originated from ancient Islamic Hadith book named Sahih Muslim). We used data scraping & web crawling with the help of Python Libraries such as Selenium automated agent-based library and Beautiful Soup to acquire the dataset with data cleaning and labelling.
Data Format	Raw: NetworkX Python Library dump Raw: Different Visualization & Exploration softwares (Gephi, Cytoscape, and Social Network Visualizer) dump Raw: Neo4J database dump Geography Markup Language format (.gml), XML-based graph format (.graphml)
Description of Data collection	The dataset used in this study was sourced from reputable repositories such as all data of Sahih Muslim with their multiple ISNAD and their narrators have been collected from Ihsan-Network website [1] and detail info of Narrator's data has been scrapped from Muslim-Scholar website [2] so that the narrator's could be assigned with the unique global identity. It was a big challenge for matching/mapping narrator's name from both two sides, at last it has been achieved by some our logical code script and some fuzzywuzzy (python) library. Finally mapped manually for remaining narrator's names which could not be mapped via automated logic. Detailed information about each dataset source is provided in Tables 1, 2 and 3. We conducted a thorough assessment to determine the potential impact of each source on the generated dataset, ensuring transparency and reliability in our data collection process. We collaborated with domain experts in Islamic studies to validate the authenticity and relevance of the dataset. The collection and processing of the dataset are based on two steps: 1. The data including the Hadith number, Narrators numbers, Sanad numbers, ISNAD wise Narrators sequence, Source Narrators & Target Narrators for each Hadith Sanad fields are collected from Hadith websites. The raw text is further processed to separate Hadith components such as Chapter, MATN, and Narrators. The resulting Chapter, MATN and Sanad fields are stored in separate columns.
Data source location	Data was gathered till December 2023, with random web crawls carried out to ensure adequate global coverage. Scraped/crawled it from https://www.ihsanetwork.org/hadith.aspx and http://muslimscholars.info/ using the Python Selenium automated agent-based library and Beautiful Soup to acquire the dataset with data wrangling and labelling.
Data accessibility	Repository name: Mendeley Data Data identification number: 10.17632/gzprcr93zn.2 Direct URL to data: https://data.mendeley.com/datasets/gzprcr93zn/2 Instructions for accessing these data: MIS contains three excel sheets (Table 1, Table 2 and Table 3)

## 1 Value of the Data

• Multi-IsnadSet is created for future research on the public repository for all the Research Institutes, Scientific and Islamic communities who want to work on Multi-ISNAD of Hadith including classification of Hadiths (Sahih=perfection in the Sanad/Dhaif=imperfection in the Sanad) [3], and narrators (trustworthy/not) [[4], [5], [6], [7], [8], [9]].
• The data can be used to build Hadith software tools for establishing social networks or graph neural network and it can become an imported source for information interaction and mapping high level relationships [[10], [11], [12], [13]]

**This dataset offers significant value and contributions to multiple research areas with categorized way:**

(1) Islamic Hadith Studies:
  • Provides a comprehensive dataset for studying Hadith narrators, ISNAD chains, and Hadith propagation in a structured graph format.
  • This data is available in the public repository for all the Research Institutes, Scientific and Islamic communities who want to work on Hadith Narrators and Hadith ISNAD.
(2) Hadith Authentication:
  • Researchers can explore the dataset to analyze the authenticity and credibility of Hadith narrators, ISNAD chains, and the propagation of Hadith in a structured graph format
  • Provides Hadith narrators and Hadith ISNAD that could be used in a wide variety of future research studies related to Hadith authentication and rules extraction.
(3) Graph Neural Networks (GNN):- The dataset is conducive to the application of Graph Neural Networks (GNNs) and offers exciting possibilities:
  • Node Embedding: GNNs can be employed to generate embeddings for each narrator, capturing their positions within the Hadith network.
  • Community Detection: GNNs can assist in the automatic detection of narrator communities based on their interactions and shared characteristics.
  • Link Prediction: GNNs can predict missing Sanad-Hadith relationships, helping reconstruct and validate the Hadith network.
  • Graph Classification: Researchers can use GNNs to classify narrators based on their attributes, authenticity, or credibility.
(4) Social Network Analysis (SNA):- This dataset offers unique opportunities for Social Network Analysis (SNA) researchers according to find Microscopic, Macroscopic, and Mesoscopic properties of Narrative Network.:
  • Network Structure: Researchers can examine the structural properties of the Hadith network, uncovering key characteristics such as network density, centrality measures, and clustering coefficients.
  • Centrality Analysis: SNA metrics like degree centrality, betweenness centrality, and eigenvector centrality can be applied to identify influential narrators and nodes within the Hadith network.
  • Community Detection: Advanced community detection algorithms can be used to group narrators with similar attributes or roles, shedding light on the organization of the Hadith network.
  • Propagation Patterns: SNA techniques can reveal how Hadith propagate through narrators, providing insights into the flow of information within the network.
(5) Neo4j Graph Database:
  • Enables researchers to test and develop graph algorithms within a well-established database environment.
  • Integration with Neo4j, a powerful graph database platform, enhances the dataset's utility:
  • Graph Algorithms: Neo4jʼs extensive library of graph algorithms can be applied to perform tasks such as finding the most influential narrators, identifying shortest paths, and detecting network anomalies.
  • Cypher Query Language: Researchers can leverage Cypher, Neo4jʼs query language, for versatile and efficient graph data retrieval, exploration, and analysis.
  • Scalability: Neo4jʼs scalability ensures that the dataset can accommodate the demands of large-scale analyses, making it suitable for comprehensive Hadith research.
(6) Cross-Disciplinary Research:
  • Encourages collaboration between scholars from Islamic studies, social network analysis, artificial intelligence and Machine Learning.
  • Facilitates interdisciplinary research, promoting transparency and reproducibility.

## 2 Background

The Hadiths, or Prophetic traditions, are narrations originating from the sayings and conduct of Prophet Muhammad (peace be upon him). Initially, the Hadiths were orally transmitted and a few decades later were committed into written form collected as small booklets. However, by the end of the second century Hijra (Islamic Calendar), scholars started compiling Hadiths into large collections classifying them subject wise. This work tackles the collection of Hadiths in Sahih of Muslim, named after Imam Muslim (d. CE 875). This collection along with that of Sahih of Bukhari (d. CE 870) are considered the most authentic collection since only the Hadiths with the most reliable transmitters were included in the collection.

The Hadith takes the form: Narr-1 → Narr-2 → Narr-3 → … → Narr-k, followed by the text of the Hadith (or, the statement of the Hadith which is also known as MATN). The ISNAD, the chain of narrators, is a reverse chronological chain of narrators involved in transmitting this particular statement. So, Narr-k is the prime narrator who personally heard the Prophet says the statement. The importance of ISNAD is that it gives credibility to the transmitted statement. Has it been transmitted exactly as said (word for word), or by meaning. To accomplish this, scholars study the history of each narrator taking into consideration, his memory, his truthfulness, etc.

This data article transformed the chains of narrations along with narrators into graphs and represented through knowledge graphs developed in Neo4j. The resultant knowledge graph will allow scientists across the disciplines to perform better analysis over structured information accordingly.

This dataset aims to provide a structured representation of MIS in Sahih Muslim Hadith, allowing for in-depth analysis of narrator chains and Hadith propagation.

## 3 Data Description

**Fig. 1:**
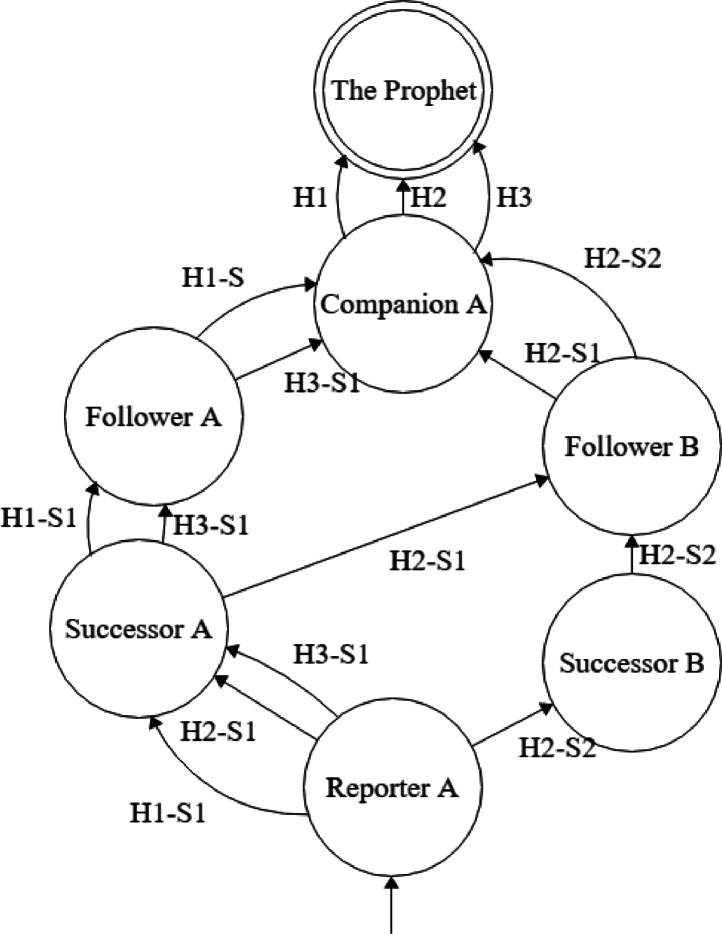
Narrator's network of three multiple transmission chain (ISNAD) of single Hadith represented using multiple edges.

The earliest work reported regarding the computational studies of Hadith was by Mustafa AlAzami [14]. A Hadith is a narrative by the Holy Prophet Muhammad (Peace be upon him). A Hadith consists on two basic constituents including the narrative and the chain of narrators. The manual curation process that performs strict analysis over a chain of narrators is termed as ISNAD (Fig. 1). The ISNAD are being evaluated throughout the chain/transmission narrators till the actual source of the narrative. The evaluation process includes the authentication of a narrator and ranking among specific classes in terms of credibility along with the thorough inspection of the continuity of the narration.

There are some qualities that are essential to determine the credibility and authenticity of Hadith in the narrators of the Hadith, which have been developed by Hadith experts around 1200 years ago. The study resulted into the advent of a new area of social studies named as Asma-ul-Rijal and Ilm-ul-Rijal (The study of authentication analysis of Hadith narrators).

This includes the narrator of the Hadith should be of firm faith, be honest, be righteous and trustworthy, have an excellent memory as well, have sound knowledge of the QURAN, HADITH and FIQAH, should report the exact words without using any word from his own. Chain continuity, integrity of narrators’ character, report precision, non-deficiency and non-aberrance are also essential for authentic criteria of Hadith.

The dataset in the article provides a valuable resource for machine learning tasks aimed at addressing the authenticity of Hadiths. For example, it can be utilized for sentiment analysis [15], classification, and clustering of Hadith [16,17]. By applying machine learning techniques to this dataset, researchers can gain deeper insights into the reliability and authenticity of various Hadiths, contributing to the broader discourse on Islamic scholarship [18].

**Fig. 2:**
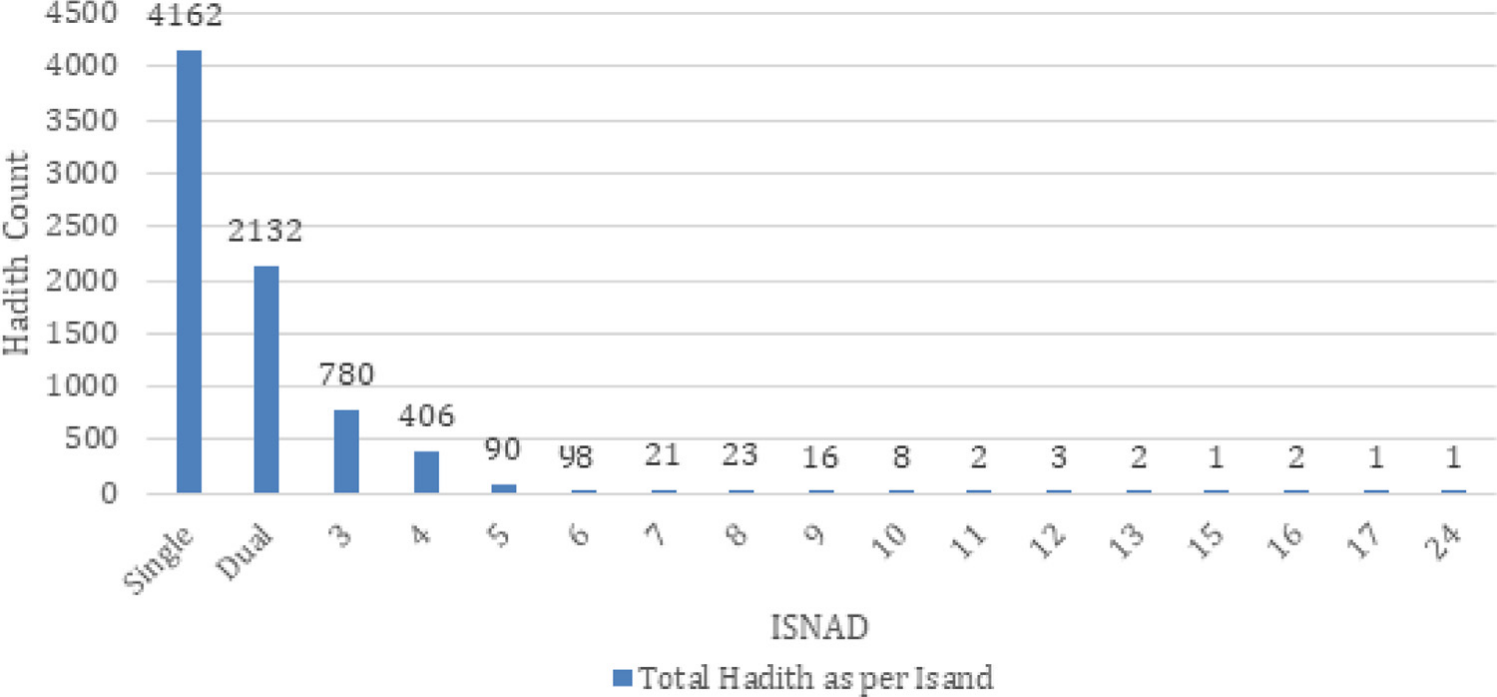
Statistical analysis Hadith according to Multiple ISNAD.

**Fig. 3:**
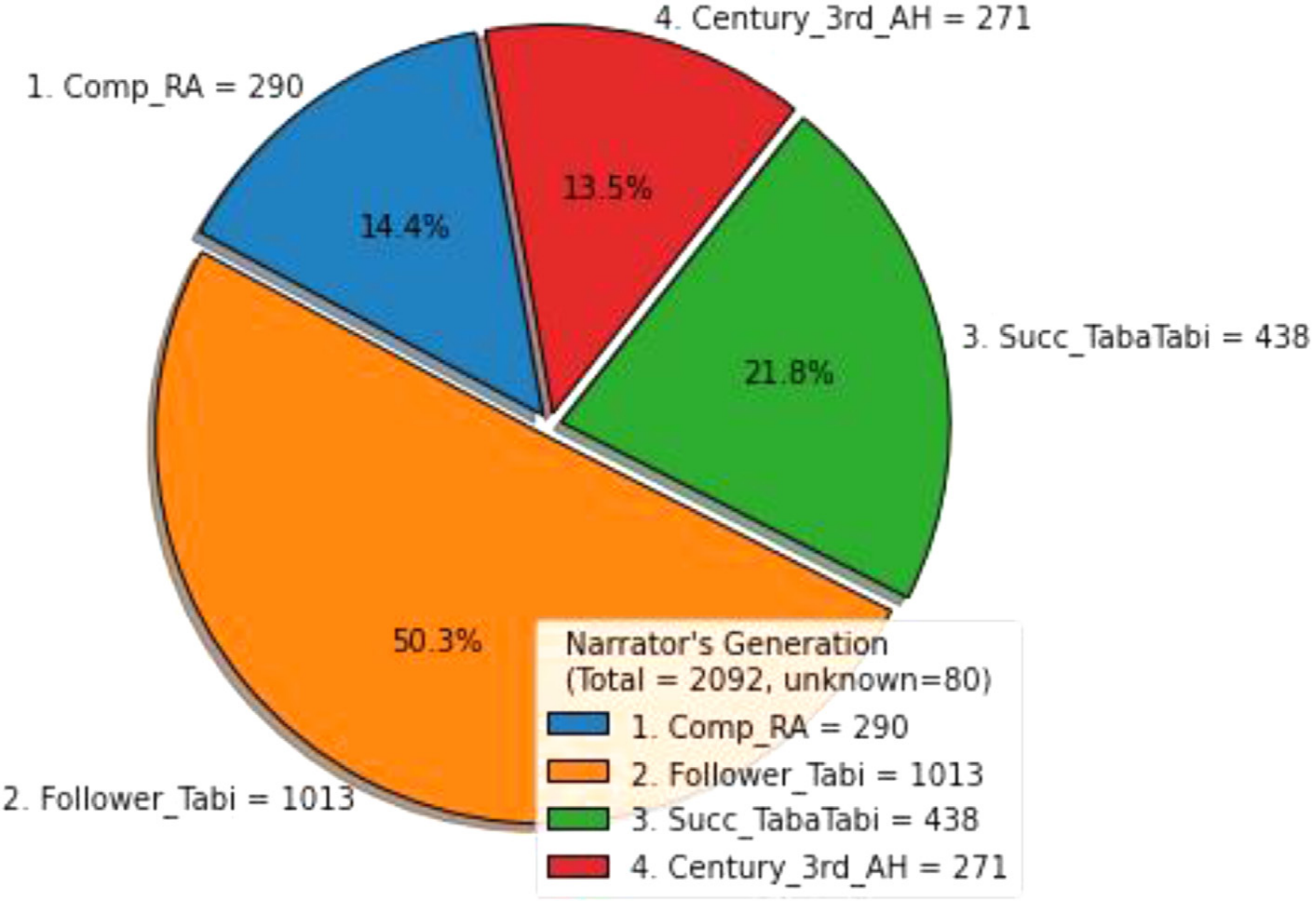
Statistical analysis for four classification of narrator's generation.

This dataset contains three excel sheets (Table 1, Table 2, Table 3): Hadith document (7748 records) and Narrators of Multi-Isnad document (77,797 records). The data contains 7748 Hadith 2092 unique records of Narrators of all Sahih Muslim Hadith. Total records of ISAND are 14,155 is presented in Fig. 2, Fig. 3:

**Table 1:** Specification for Dataset File (1_Hadith_SahihMuslim_HadithContent.xlsx).

Seq. No	Attribute	Description	Example Value
1	BookNo	Global ID of the Hadith Book	2
2	BookTitleEnAr	Book Title with English and Arabic Text	Sahih Muslim - الصحیح المسلم
3	HadithNo	Unique Hadith No of the particular Book	1
4	ChapterNo	Unique Chapter No of the particular Book	1
5	ChapterTitleAr	Chapter Title with Arabic Text	المقدمة
6	ChapterTitleEn	Chapter Title with English Text	Introduction - كتاب المقدمة
7	UnitNo	Unique Unit No of the particular Chapter	1
8	UnitTitleAr	Unit Title with Arabic Text	باب وجوب الرواية عن الثقات وترك الكذابين .
9	HadithText_Mushakkal	Arabic Hadith Content with Diacritize text	‏"‏ مَنْ حَدَّثَ عَنِّي، بِحَدِيثٍ يُرَى أَنَّهُ كَذِبٌ فَهُوَ أَحَدُ الْكَاذِبِينَ ‏"‏
10	HadithText_GhairMushakkal	Arabic Hadith Content without Diacritize	‏"‏ من حدث عني، بحديث يرى أنه كذب فهو أحد الكاذبين ‏"‏
11	SanadCount	Sanad Count of the particular Hadith	3
12	SanadWithNarratorsCount	e.g. 3 ISNAD & 6 Narrators for each Sanad	1 = 6,2 = 6,3 = 6

**Table 2:** Specification for Dataset File (2_Hadith_SahihMuslim_Narrators=Nodes for Graph.xlsx).

Seq. No	Attribute	Description	Example value
1	NarratorID_Mapped	Global Narrator ID for Node Unique ID	2
2	NarratorNameAr	Narrator Complete Name in Arabic Text	عبد الله بن عثمان أبو بكر الصديق
3	NarratorNameAr-Mapped	Narrator Short Name in Arabic Text	أبو بكر الصديق
4	NarratorNameEn-Mapped	Narrator Name for Node Label Property	Abu Bakr As-Siddique (0)
5	Gender	Narrator Gender for Node property	Male
6	NarratorGeneration	Narrator Generation for Node property	Comp.(RA)

**Table 3:** Specification for Dataset File (3_Hadith_SahihMuslim_Isnad=Edges for Graph.xlsx).

Seq. No	Attribute	Description	Example value
1	BookNo	Global ID of the Hadith Book	2
2	HadithNo	Unique Hadith No of the particular Book	1
3	ChapterNo	Unique Chapter No of the particular Book	1
4	UnitNo	Unique Unit No of the particular Chapter	1
5	SanadNo	Unique Sanad No of the particular Hadith	1
6	sourceNarratorID	Source Node ID for Directed Graph	166
7	sourceNarratorNameEn	Source Node property	al-Mughira ibn Shu'ba (0)
8	targetNarratorID	Target Node ID for Directed Graph	10,834
11	targetNarratorNameEn	Target Node property	Maymun bin Abi Shbyb
12	intractionLabel	Complete info of Single Hadith's SANAD for Edge property	B2-H0001-C01-U01-S01-i1

The first major generation records result is Follower_Tabi with 1013 Narrators (50.3 %) and Succ_TabaTabi, Comp_RA and Century_3rd_AH ranked second, third and fourth with 438 Narrators (21.8 %), 290 Narrators (14.4 %) and 271 Narrators (13.5 %) respectively. Fig. 3 shows the generation's categories according Sahih Muslim Hadith book.

**Fig. 4:**
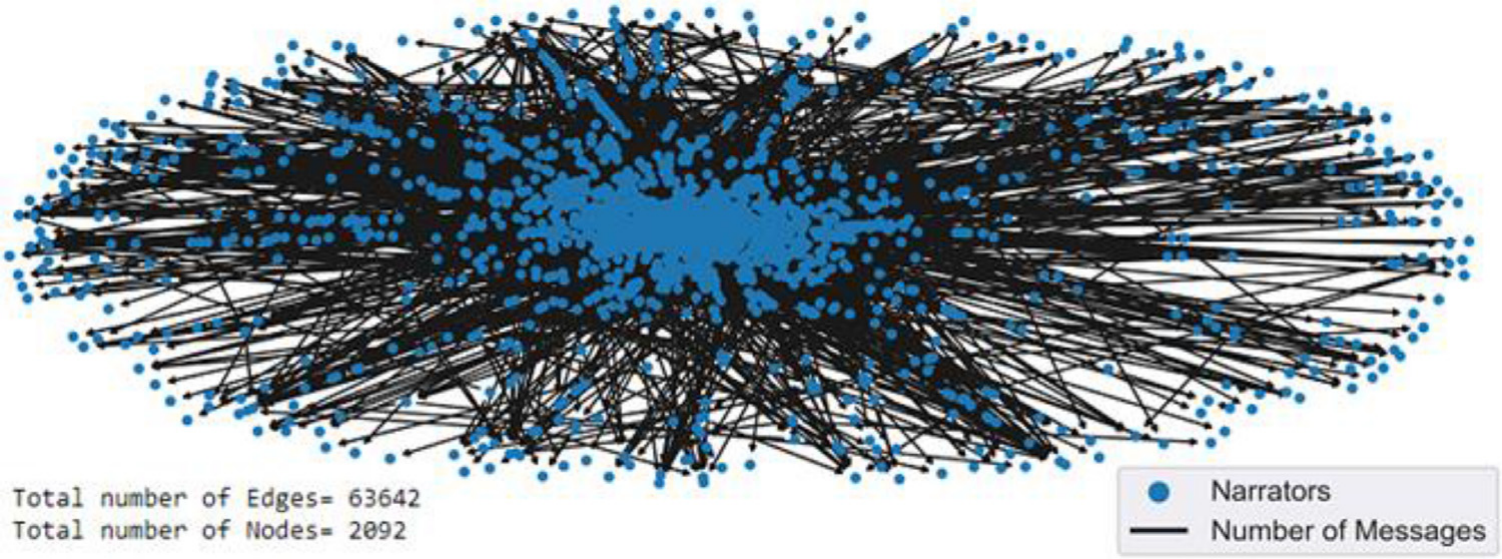
Narrator's network with multiple ISNAD represented by Python LIBRARY.

A complete picture of the Global Hadith SANAD Narrator's Network from the book Sahih Muslim with 2092 total number of Narrators (nodes) and 63,643 total number of their Transmission (edges) has been shown in Fig. 4.

## 4 Experimental Design, Materials and Methods

Data Collection and Preprocessing: The dataset's creation followed a systematic approach:

• Data Extraction: Information was sourced from Islamic texts, Hadith databases, and authoritative references. Data extraction focused on collecting Hadith text, ISNAD chains, and narrator details.
• Data Transformation: The collected data underwent transformation to represent narrators as nodes and ISNAD-Hadith relationships as edges in the graph structure.
• Quality Control: Stringent quality control checks were implemented to ensure the dataset's accuracy. This included standardizing naming conventions, resolving discrepancies, and addressing any missing or erroneous data points.

Multi-IsnadSet Statistics: The dataset's statistics include:

• Nodes (Narrators): 2092 individual narrators.
• Edges (Sanad-Hadith Relationships): 77,797 relationships representing the propagation of Hadith through narrators.

The data is crawled from multiple web sources (that are manually curated), then data was pre-processed (cleaning, removing stop-Word, normalizing Arabic letter etc.) and merged. A few mismatched narrator's names are found from different resources. The mismatch issue is resolved by different logical code script and fuzzywuzzy (python) library. The study attempted to match the complete name of narrators mentioned in Hadith while normally transmitter's full name is not mentioned in given Hadith. The transmitters who have similar names in context of chain of narrators are further investigated. All unique chains of narrations that reports the same Hadith with little variation of words are identified and reported. Similarly, all different chains of single Hadith are identified and mentioned separately having a variation in narrator's chain. The social network of narrators and chain of narrators is represented through a graph G(V, E) where vertices represents the narrators and edges represented the narrations. Multiple tools are employed in representation, analysis and database for graphs including Python Library (NetworkX), Graph Database Neo4j and two different network analysis tools named Gephi and CytoScape [Table 4].

**Table 4:** Narrator's network with multiple ISNAD including single Hadith represented by three different network tools (Neo4j – Gephi – CytoScape).

## Limitations

Regarding the limitation section, the following points are included:

• Data Limitations: Data has been collected exclusively from the Hadith book named Sahih Muslim only.
• Domain or Context Limitations: The dataset primarily focuses on Hadith authentication and analysis, limiting its applicability to broader religious or cultural contexts outside of Islamic scholarship.
• Incomplete Information of Narrator: The dataset may lack comprehensive details about narrators, including their stay locations, traveling locations and generation/level of narrators

## Ethics Statement

This data is available in the public domain, and no funding is received for the present effort. There is no conflict of interest.

The authors state that this work involved:

- No human subjects.
- No animal experiments.
- No data collection from social media platforms.

Copyright: Data is from public domain; it is dated to decades and centuries. The data does not belong to users on the web resource (i.e., social media). The data is published on free and public Islamic websites and is available to anyone with internet access.

Privacy: While the data is free and public, we anonymize the website and Hadith pages.

Scrapping policies: The web resource does not have any special scrapping policy*.*

## CRediT Author Statement

**Aziz Mehmood Farooqi**: Conceptualization, Methodology, Data curation, Formal analysis, Software, Validation, Investigation, Writing- Reviewing and Editing **Rauf Ahmed Shams Malick**: Supervision, Conceptualization, Writing-Original Draft, Writing- Reviewing and Editing **Muhammad Shahzad Shaikh**: co-supervision, Domain Expect, Validation, Writing- Reviewing and Editing **Adnan Akhunzada**: Writing- Reviewing and Editing.

## Data Availability

Multi-IsnadSet MIDS for Sahih Muslim Hadith with chain of Narrators, based on multiple ISNAD (Original data) (Mendeley Data).
